# Syphilis

**Published:** 1877-07

**Authors:** Ferdinand King

**Affiliations:** Atlanta, Ga.


### SYPHILIS.

By FERDINAND KING, M.D., Atlanta, Ga.

(*Read before the Atlanta Academy of Medicine.*)

This disease has puzzled medical writers through almost four centuries past, and still stands as a stumbling-block in the way of the most scientific of our profession. Even Hippocrates, Celsus, and other writers of their day dwelt at length upon its history, diagnosis, treatment, etc., but no two of these agree in toto upon the main points referred to in its discussion.

Its origin and history have been studied more attentively of late years than in the days of those writers just referred to, but in my opinion it is even now an unsolved problem. Being an advocate of that doctrine—however paradoxical it may seem—that every man has a right to his own honest opinion, I feel myself in no way bound to accept as true everything that is printed in our standard medical works; therefore I propose to advance an idea of my own in regard to the origin and history of syphilis. This may seem presumptuous in me, one whose medical sun is hardly above the eastern hilltops; still I plunge into the unfathomed depths, promising myself your indulgence in my efforts to reach a safe port. I claim no originality for any thing I may write, as I agree with the old adage, "There is nothing new under the sun." No discovery or invention is ever made perfect, or theory permanently established, by the originator thereof. Our ideas upon all things—whether physical, chemical, or medical—though they may at a glance seem original, are found, on closer investigation, to consist of the suggestions of others, perfected or made more available. Thus Jenner learned the secret of vaccination from his observations among the milkmaids of his time. Esmarch's bloodless amputation was suggested by the sight of a dying soldier, who had stopped the flow of his life blood by binding his arm tightly with his blanket strap. Brown-Sequard perfected his plaster of Paris apparatus or splint after seeing a similar contrivance made by a backwoods medical student whose name is unknown. And so with all other discoveries or inventions in our profession as well as in the arts. Hence any theory I may advocate or suggest, is the result of reading the writings of my predecessors.

In regard to the origin of syphilis, I have found no authority with which I agree. Bumstead and several others equally prominent in venereal, seem to think the disease originated about the close of the fifteenth century, or at the time of the invasion of Italy by the French army under Henry VIII.—these soldiers carrying the disease on this campaign. French writers defend their countrymen, and say the disease was unknown in France prior to this date, which was the years 1493 and 1494. Other writers, entitled to equally as much credit, assert that it was first carried to Europe by the crew of Columbus, on his return from his first voyage to the West Indies, in 1493. That the disease existed in the new world at the time of Columbus' second voyage, is a well established fact. As the discovery of America and the invasion of Italy by Henry VIII. took place simultaneously, and the friends of these countries—France, Italy, and America—defend their respective sections against the origin of this loathsome disease with Spartan courage, all our leading syphilographers seem to make no efforts to unravel the mystery prior to the time referred to. In other words, they decline, for some unknown reason, to go behind these ancient returning boards.

I believe that syphilis is almost or quite as old as history itself; that even the sons of Abraham and Isaac were not strangers to it; that it was intended by our Creator as a moral protector, as no man or woman ever was known to have the disease unless they had violated some law of morality. Even Moses laid down rules for the government of those afflicted with the disease, as is clearly shown in the 15th chapter of Leviticus. Though Bumstead traces the history of gonorrhœa to this same chapter, I claim the right to disagree with the learned venerealist in so far as appertains to the 32d verse of this chapter, treating of the subject of sores or running issues. The great law-giver closes by saying: "This is the law of him that hath a running issue, and of him whose *seed* goeth from him and is *defiled* therewith." As no one will pretend to say the seed of man is defiled by gonorrhœa, I think all will agree that reference is plainly established. Again, it is referred to in the 5th verse, 22d chapter of Exodus, part of which speaks of "visiting the iniquity of the father upon the children, even unto the third and fourth generations." I think the disease of olden times, referred to as lepra, was certainly tertiary syphilis, as this disease is spoken of in immediate connection with clap in 32d chapter Leviticus: "What man soever of the seed of Abram is a leper or hath a running (sore or) issue, he shall not eat of the holy things until he be clean." I could quote numerous other biblical facts to prove that the disease under consideration existed long before the Christian era (but known by a different name), did the limits of this paper permit. As the Almighty intended that "like should seek like," and that each nationality should multiply within itself, he visited this curse upon those who violated his laws, in the most remote ages.

Now, to give my theory in regard to the history or transmission of syphilis from generation to generation. I must be permitted to suggest an opinion that will no doubt meet the universal condemnation of this Academy—perhaps deservedly so, as I am not by any means authority on the subject. That opinion is this: that the virus of a chancroidal ulcer, when inoculated or transferred from one subject to another—Irish or German, for example—of different stock or nationality, produces pure, unadulterated syphilis. In other words, I claim that what is ordinarily known as diagnosticated chancroid is simply a mild form of syphilis. This may seem old time, nevertheless I think historical as well as common sense facts render the opinion worthy of credit. The disease (syphilis) when left among people of the same blood, and with the same temperament, disposition, etc., naturally tends to self-limitation or degeneration; just as intermarriages among members of the same family are followed by idiots and deformed offspring. No doubt the Jews, in the days of Abraham and Isaac, had syphilis, which, after coursing through the veins of several generations of their people, became almost inert, or in other words, degenerated into a local contagious ulcer of the genitals. As those people began to form new republics or governments at a distance from each other, a change of habits, temperament, etc., was the result, which soon made the different sections produce people entirely new to each other, thus furnishing new material or food for this treacherous disease. Then Europe was peopled. Some time after this the disease raged with renewed energy there. By the time Henry VIII. invaded Italy, the disease no doubt had become so weak that it was simply local. I expect the Italians were also subject to a mild form of it at the time Henry VIII. visited them. Being entirely a different people, syphilis soon found fresh material from which to derive new and more inflammable fuel for the flame to rekindle upon. I believe our authorities admit that chancroid or local syphilis existed both in Spain and France at this time (1493 and 1494). It is reasonable to suppose, then, that Columbus' crew carried their time-honored friend, chancroid, with them on their first voyage of discovery to the new world. As soon as they communicated it to the Indian there, it found rich, pure, warm blood from which to derive the necessary food for a regeneration. Consequently, upon the return of Columbus on a second voyage to the West Indies, he found syphilis "blooming like a morning rose." Recent rèports from the surgeons in and around Philadelphia go far towards sustaining me in my theory. These physicians say that syphilis of a more serious character than ever before seen there prevails to an alarming extent since the Centennial. This I attribute to the visit from our trans-Atlantic brethren during the international exhibition, when Russians, Turks, Egyptians, Malays, and every known nationality under the sun mixed freely with our American people. You who have been called upon to treat syphilis among the newly-arrived Jews and Italians, fresh from their native country, will, I think, readily agree that it is more troublesome than when found among our own citizens.

But as I have already, I fear, given too much space to the origin and history of the disease, I must hasten on to its diagnosis, prognosis, and treatment. Of the former I need say but little, as you have all seen enough of it to become even more familiar with it than myself. The only point that requires *careful* attention and good judgment is in deciding whether to pronounce the ulcer to be the result of a poison that is strong enough to produce a constitutional effect, or is merely a local solution of continuity; or in other words, to say whether the disease is chancroid or chancre. This can easily be determined by inoculation—the former producing only a simple ulcer similar to the one from whence came the matter, while the latter produces little or no effect.

Syphilographers, for the sake of convenience, divide the disease into three stages—primary, secondary, and tertiary. We hardly ever, in my judgment, see a case of primary syphilis; and when we do, the symptoms are so obscure as to generally deceive us. These symptoms consist of depression of spirits, lassitude, or hypochondria, and more or less headache, confined principally to the cervical vertebra and occipital region. The patient at the same time suffers from constipation and heartburn. In this stage, I say, the patient seldom consults the physician. It is not until the chancre makes its appearance that he seeks medical aid. Then we have to deal with secondary or constitutional syphilis, even though the poison was inoculated upon a raw or abraded surface, for the blood makes its round through the entire system in a very few minutes, thus carrying the septic matter (for as such I regard this specific virus) to every vital part of the human subject. When this poison is so far removed from the original, or last fresh subject from whence it came as to become almost inert, then it is too weak to interfere with or overcome the efforts of nature in her own defense, save in or upon the genitals, where the weakest points are found; hence in these cases we find only local lesions or chancroid, yielding readily to direct or local treatment. And here I would remark, per parenthesis, that it is a good idea to always question your patient very particularly in regard to his general health and feelings during the few days preceding the time of his first consultation with you, or the first appearance of the ulcer he presents. If he has observsd the headache, etc., as above referred to in symptoms of the primary stage, then you may safely pronounce the case one of constitutional or secondary syphilis. If you find that you cannot determine the nature of the disease by these inquiries, I see no harm that could possibly arise from the adoption of a constitutional treatment anyway, even though it may be merely a local or mild form of the disease.

If your patient is not inclined to deceive you by false representations, there is no trouble in diagnosticating tertiary syphilis, as it has certainly been preceded in nine-tenths of cases applying for treatment by primary and secondary symptoms. Then the peculiar symptoms of this stage of the disease are so marked and easily defined as to make their diagnosis quite easy. Syphilitic rheumatism, alopœcia, blotches, or papulæ upon the skin, chronic hard engorgement of the post cervical glands, as well as the inguinal, chronic or ulcerated sore throat, and general or perpetual depression of spirits are nearly always plainly observable. Even with all these the patient sometimes succeeds in deceiving the most expert surgeons. A man who has syphilis will tell lies on this one thing when his veracity on every thing else is unimpeachable. Sometimes we find our patients in this third stage, confirmed syphilomaniacs.

Of the prognosis of syphilis I have but little to say, from the fact that I have not been engaged in the practice of medicine a sufficient length of time to justify a fixed opinion. Although there has been no recurrence of the disease in patients treated four or five years ago, I have no evidence to prove that it will not break out anew at an early day. Whether a physician is justifiable in promising his patient a permanent cure is a question with rather an uncertain answer, in my mind. My plan is to cheer him up as much as possible. Sometimes I find it necessary to promise him a radical cure, thus stimulating nature in defense of her own domicil. I see no good reason why syphilitic virus may not be entirely eradicated from the human system in the same way that other septic material is expelled. Nature is a powerful adjuvant or co-worker in accomplishing such a purpose—expelling virus, as well as all other septic material, as her most uncompromising enemies; an arch fiend that should be like unto the seven devils that were so unceremoniously cast out of Magdalene of old. Perhaps we do not at present possess those remedies capable of accomplishing this purpose. If not, I think our profession, aided in the work by chemical and microscopical investigations, will come into possession of them at an early day. We must expect material aid from the intelligent physiologist, whose duty it is to familiarize the student with the chemical constituents of the various tissues that enter into the make-up of man.

We next come to the treatment of syphilis. The first thing to be spoken of in connection with this part of my subject is hygiene, without proper attention to which we cannot promise success to ourselves or our patients, however favorable or unfavorable the case may seem. The most important features in connection with the hygiene are *system* and the adoption of those sanative measures usually regarded so essential to the successful treatment of all disaeses arising from the presence of septic material, that invariably produces a depraved condition of that great life-giving and life-supporting fluid, the *blood*. The physician should try to impress upon the mind of his patient the importance of *regularity* in all his habits of life. He should have simple but nourishing food; abstain from the use of tobacco and whisky. If he is compelled from force of habit to have a stimulating beverage, an occasional glass of ale or porter may be permitted. The functions of the skin, kidneys, bowels, etc., should be carefully attended to, as these are the great avenues through which nature, aided by art, expels her enemies. He should take his meals regularly, sleep at least seven out of every twenty-four hours, avoid all sedentary habits, taking plenty of out-door exercise, and control his sexual desires as far as possible. This latter precaution should be carefully looked after, as syphilitics, like consumptives, oftentimes imagine the end is near; hence plunge into excessive venery. Hence the importance of endeavoring at all times to keep up a cheerful disposition.

The medicinal agents employed in the treatment of syphilis are numerous. Space will not permit me to speak of them separately at length; therefore I shall restrict myself to the consideration of those most usually recommended and employed. Mercury has for centuries past been regarded the sheet anchor in the treatment of syphilis. The use and abuse of this remedy alone would furnish material enough for a considerable volume. I am satisfied that the remedy is too often used empirically, from the fact that many who employ it frequently have very little idea of its specific effects. Some use it because their preceptors or "medical daddy" thought it cured all forms of syphilis, while others think there is no chance for improvement on the old dogma. With all due respect for my preceptor, who was a "mercurio-maniac," conscience prompts me to ignore his teachings, in so far as this agent is concerned. I am satisfied that we can treat the disease more successfully by the use of other less objectionable remedies. Mercury, we all know, when employed for any considerable length of time, acts as a depletory; it smacks smartly of homœopathy, I think, to employ it where every symptom calls for a tonic treatment. Instead of this remedy, the use of which is truly time-honored, I in all stages of the disease employ iodine, in combination with potassa and vegetable alteratives. The following prescription, I think, meets a majority of the symptoms usually met with in syphilis:

℞.—Pot. Iodide, . . . . . grs. 320
Ferri Phos., . . . . . grs. 192
Syr. Stillingia, . . . . .
Syr. Helonias, . . . . . aa. f℥ij
Tr. Phytolac Dec., . . . . ℥ss
Elixir Simplex, . . . . . ℥viii
M. ft. sol.

Dose, teaspoonful three times a day before meals.

In this combination we have an alterative, tonic, diuretic, and aperient, which is surely calculated to repel every attack made upon any organ by this most deceptive of all diseases. All the cases I have had got along finely under this treatment. I think the medicine should be continued at least eighteen months; in fact, I always advise my patients to continue it for two years, changing the prescription from time to time so as to meet the different pathological indications as they present themselves. By the use of these remedies thus combined we keep the bowels in a soluble condition, the liver slightly stimulated; the iron keeps the blood up to the healthy standard, supplying red corpuscles, etc. Nature thus aided is, I think, capable of freeing the system from the effects of this deadly poison in the time above specified. Long years of experience will either strengthen me in this belief or scatter to the winds every vestige of faith in my imaginary "eureka."

It would not be out of place, perhaps, to say a few words here upon the treatment of venereal ulcers. After thoroughly cauterizing with hydrarg. nit. any ulcer that makes its appearance upon the genital organs, I apply either a dry or an emollient dressing. The former is usually composed of equal parts of calomel and hydrastis sulphas; the latter simple cerate with carbolic acid or ung. hydrarg. nitras.

In conclusion, I would say that I am inclined to believe that syphilis could be entirely expelled or eradicated from the human family by the adoption of certain sanitary laws and regulations. These should be more rigidly enforced in the seaport cities, where foreigners more frequently and *first* come together, as I am sure the disease is increased in virulence only by adding fresh fuel to the flame. Remove the virus two or three generations from its origin or newly acquired fountain head, and it is almost harmless. I however think a little syphilis a good thing to have around, as a moral agent, when we can confine it to certain classes. But I must refer the discussion of this part of my subject to those engaged in another field. I thank you, Mr. President and gentlemen of the Academy, for your kind attention.

